# Preservation and Significance of Extracellular DNA in Ferruginous Sediments from Lake Towuti, Indonesia

**DOI:** 10.3389/fmicb.2017.01440

**Published:** 2017-07-27

**Authors:** Aurèle Vuillemin, Fabian Horn, Mashal Alawi, Cynthia Henny, Dirk Wagner, Sean A. Crowe, Jens Kallmeyer

**Affiliations:** ^1^GFZ German Research Centre for Geosciences, Section 5.3: Geomicrobiology Potsdam, Germany; ^2^Research Center for Limnology, Indonesian Institute of Sciences Cibinong-Bogor, Indonesia; ^3^Department of Microbiology and Immunology, University of British Columbia, Vancouver BC, Canada; ^4^Department of Earth, Ocean, and Atmospheric Sciences, University of British Columbia, Vancouver BC, Canada

**Keywords:** extracellular DNA, intracellular DNA, environmental archive, cell lysis, GC content, ferruginous sediment, Lake Towuti, ICDP drilling

## Abstract

Extracellular DNA is ubiquitous in soil and sediment and constitutes a dominant fraction of environmental DNA in aquatic systems. In theory, extracellular DNA is composed of genomic elements persisting at different degrees of preservation produced by processes occurring on land, in the water column and sediment. Extracellular DNA can be taken up as a nutrient source, excreted or degraded by microorganisms, or adsorbed onto mineral matrices, thus potentially preserving information from past environments. To test whether extracellular DNA records lacustrine conditions, we sequentially extracted extracellular and intracellular DNA from anoxic sediments of ferruginous Lake Towuti, Indonesia. We applied 16S rRNA gene Illumina sequencing on both fractions to discriminate exogenous from endogenous sources of extracellular DNA in the sediment. Environmental sequences exclusively found as extracellular DNA in the sediment originated from multiple sources. For instance, *Actinobacteria, Verrucomicrobia*, and *Acidobacteria* derived from soils in the catchment. Limited primary productivity in the water column resulted in few sequences of *Cyanobacteria* in the oxic photic zone, whereas stratification of the water body mainly led to secondary production by aerobic and anaerobic heterotrophs. *Chloroflexi* and *Planctomycetes*, the main degraders of sinking organic matter and planktonic sequences at the water-sediment interface, were preferentially preserved during the initial phase of burial. To trace endogenous sources of extracellular DNA, we used relative abundances of taxa in the intracellular DNA to define which microbial populations grow, decline or persist at low density with sediment depth. Cell lysis became an important additional source of extracellular DNA, gradually covering previous genetic assemblages as other microbial genera became more abundant with depth. The use of extracellular DNA as nutrient by active microorganisms led to selective removal of sequences with lowest GC contents. We conclude that extracellular DNA preserved in shallow lacustrine sediments reflects the initial environmental context, but is gradually modified and thereby shifts from its stratigraphic context. Discrimination of exogenous and endogenous sources of extracellular DNA allows simultaneously addressing in-lake and post-depositional processes. In deeper sediments, the accumulation of resting stages and sequences from cell lysis would require stringent extraction and specific primers if ancient DNA is targeted.

## Introduction

Extracellular DNA (eDNA) is ubiquitous in sediment and soil (Pietramellara et al., 2009) and often comprises the largest fraction of total environmental DNA (Ceccherini et al., 2009). In aquatic systems, it consists of genetic material derived from surrounding soils, water, and underlying sediments. It includes nucleic acids from damaged dead cells, released upon lysis or actively excreted into the surrounding water and sediment (Torti et al., 2015). This fraction is partitioned between sorption onto mineral matrices (Crecchio et al., 2005; Cleaves et al., 2011) and degradation via microbial metabolism (Corinaldesi et al., 2007). Adsorption promotes its persistence, and deposition and burial in sediments, whereas free DNA is readily available for microbial uptake as a nutrient source, resulting in variable turnover rates of this fraction (Dell’Anno and Corinaldesi, 2004; Corinaldesi et al., 2008). Dell’Anno and Danovaro (2005) suggested that eDNA plays an important role in biogeochemical element cycling in the subsurface. However, the decrease of microbial activity as a function of increasing depth below the water sediment interface (Berner, 1980; Horsfield and Kieft, 2007) can also result in substantial release of nucleic acids into the surrounding sediments due to cell lysis or active excretion by living cells (Levy-Booth et al., 2007; Carini et al., 2016).

In theory, sedimentary eDNA should be composed of ancient and extant microbial nucleic acids with different degrees of preservation recording microbial processes formerly occurring on land, in the water column and sediment (Torti et al., 2015). Metal oxides and colloid minerals have the potential to adsorb eDNA in soils of the catchment, in the water column and/or in the sediment and cause it to persist after burial (He et al., 2008; Ceccherini et al., 2009; Cleaves et al., 2011). As long as it is preserved in the sediment, eDNA sources could represent genetic archives of past environments and processes at different points in time (Corinaldesi et al., 2011; Torti et al., 2015). However, metal-reducing microbial communities, which can use ferric iron phases as terminal electron acceptors, directly or indirectly modify the sediment sorption capacity (Dong et al., 2003; Crowe et al., 2007b). The sediment-bound eDNA is thus not entirely protected from remobilization and degradation (Baldwin, 2013) as it could be liberated and subject to microbial uptake after deposition (Dell’Anno and Danovaro, 2005; Corinaldesi et al., 2008).

Genetic material directly extracted from environmental samples without obvious biological sources is increasingly employed to study organisms that live or lived in the catchment, water column or sediment (Coolen et al., 2004, 2013; Boere et al., 2009; Ariztegui et al., 2015; Thomsen and Willerslev, 2015; Parducci et al., 2017). In ancient records, it is often best preserved within intact resting stages (Domaizon et al., 2017), such as pollens, planktonic cysts or endospores, which can then be used to trace past environmental changes (Coolen and Overmann, 1998, 2007; Parducci et al., 2005; Coolen et al., 2013) and complement other molecular indicators (Coolen et al., 2008, 2009; Boere et al., 2009; Vuillemin et al., 2014b, 2016a). However, persistent activity of microorganisms in sediments also alters this record due to selective preservation of previous assemblages (Boere et al., 2011a,b). The extent to which the extracellular fraction included in the total sedimentary DNA is an effective archive of environmental history remains untested across the vast diversity of freshwater ecosystems. In addition, the presence of multiple sources of eDNA in the sediment tends to undermine interpretations of the living subsurface biosphere in terms of abundance and diversity (Luna et al., 2006; Alawi et al., 2014; Carini et al., 2016).

As the concentration, preservation and composition of eDNA in lacustrine sediment depend on a complex interplay of processes and timing, the reconstruction of past and present microbial communities must be made within the context of these factors. To trace the origin and composition of eDNA and test its capacity to act as an archive of lacustrine conditions, we sequentially extracted extra- and intracellular DNA (iDNA) from sediments of ferruginous Lake Towuti, Sulawesi, Indonesia (Lehmusluoto et al., 1995).

### Study Site

**FIGURE 1:**
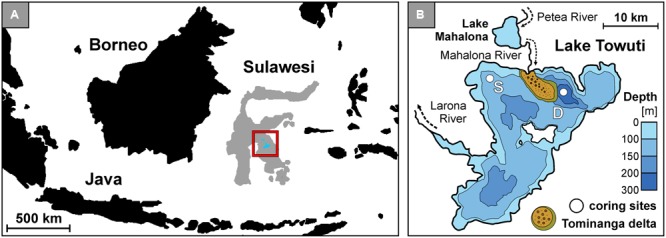
Lake Towuti location, bathymetric map with the Tominanga delta and two drilling sites. **(A)** Map of the Indonesian archipelago displaying the location of Sulawesi Island and Malili Lake System. **(B)** Bathymetric map of Lake Towuti (after Russell et al., 2016) with its major tributary and river delta (after Vogel et al., 2015), with locations of the shallow (S: 60 m water depth) and deep (D: 200 m water depth) coring site.

Lake Towuti (2.5°S, 121°E) is a tropical 200 m deep lake located on Sulawesi Island, Indonesia (**Figure 1A**). It is the largest (560 km^2^) of five interconnected lakes constituting the Malili Lakes system (Haffner et al., 2001). The Malili Lakes system is seated in a tectonic basin formed by strike-slip faults (Kadarusman et al., 2004) surrounded by ophiolitic rocks and lateritic soils (Lehmusluoto et al., 1995; Russell et al., 2016). The Mahalona River, which is the main inflow to the north of Lake Towuti, drains the catchments of Lake Matano and Lake Mahalona (**Figure 1B**), while the Larona River constitutes the only outflow to the west (Vogel et al., 2015). The tropical climate and lateritic weathering of the (ultra)mafic catchment cause strong iron fluxes with a dearth of sulfate to the lakes (Crowe et al., 2004; Golightly, 2010), exerting a decisive constraint on bioavailable phosphorus in the epilimnion as it is scavenged by iron oxy(hydr)oxides and goethite from soils (Crowe et al., 2007b; Katsev et al., 2010). This likely drives the Malili Lakes toward severely nutrient-limited conditions and restricted primary productivity (Bramburger et al., 2008; Zegeye et al., 2012).

However, iron reduction in the anoxic hypolimnion of stratified Lake Matano leads to the partial release of adsorbed phosphorus into the water (Crowe et al., 2008b). Lake Towuti’s water column is also presently oxygen-depleted below ca. 130 m depth, but unlike upstream Lake Matano which is permanently stratified and anoxic below 110 m water depth (Crowe et al., 2008b; Katsev et al., 2010), Lake Towuti is reported to mix entirely at least occasionally (Haffner et al., 2001), presumably causing transient bottom water oxygenation (Costa et al., 2015). Otherwise, its water column is slightly alkaline (pH = 8.4 to 7.2) and weakly thermally stratified (i.e., 31–28°C) (Nomosatryo et al., 2013). Due to the scarcity of sulfate in the system (<20 μM), the bottom water is anoxic but non-sulfidic and thus represents an iron-dominated ecosystem displaying dynamic redox conditions (Vuillemin et al., 2016b). The sediment deposition rate is of ca. 2 mm year^-1^ (Vogel et al., 2015) with detrital iron inputs to the sediment mostly consisting of goethite (α-FeOOH) and ferrihydrite (Fe_2_O_3_.0.5H_2_O) (Crowe et al., 2004). Through adsorption these ferric iron phases could promote the preservation and persistence of multiple sources of eDNA in the sedimentary record of Lake Towuti since the late Pleistocene (Russell et al., 2016).

## Materials and Methods

### Sample Processing

In autumn 2013, several short sediment cores (<0.5 m) estimated to cover ca. 1750 years of sedimentation history (Vogel et al., 2015) were retrieved at a shallow (60 m water depth) and deep site (200 m water depth) (**Figure 1B**). The two sites displayed oxic and anoxic conditions in the bottom water, respectively (Vuillemin et al., 2016b), with oxygen penetration in the sediment limited to less than 2 mm at the shallow site (data not shown). In order to prevent oxidation of the sediment, entire short cores were sampled inside a glove bag flushed with nitrogen gas and aliquoted for multiple measurements using a sterile spatula cleaned with absolute ethanol after each use. For nucleic acid analyses, cores were sectioned into 1, 2, and 5 cm intervals and sediment samples were packed into gas-tight aluminum foil bags flushed with nitrogen gas and heat-sealed to keep them under anoxic conditions. The samples were stored at room temperature (25°C) for 5 months until DNA extraction in the home lab. General geochemical data were presented and discussed in detail in a previous study (Vuillemin et al., 2016b). The corresponding dataset is archived on the Pangaea^®^ database^1^ under accession number # 861437 (Vuillemin et al., 2016c).

### Extra- and Intracellular DNA Extraction

Commonly, DNA is extracted from soil or sediment through a total DNA (tDNA) extraction approach without taking into account that the extractable DNA pool consists of iDNA and eDNA (Corinaldesi et al., 2005; Levy-Booth et al., 2007; Ceccherini et al., 2009). Separating these two fractions during the extraction procedure allows for a more specific analysis of the extant microbial communities with intact cell walls and the ancient communities preserved as eDNA.

The protocol of Alawi et al. (2014) was applied for separate recovery of eDNA and iDNA from a single sample. All extractions were performed in duplicates along with a negative control. In brief, we mixed 1.0 g of fresh sediment with 0.2 g of acid washed polyvinylpolypyrrolidone (PVPP) and 2.5 mL of 0.1 M sodium phosphate buffer (Na-P-buffer). Sample slurries were centrifuged and their supernatants carefully decanted off. The procedure was repeated twice and supernatants were pooled to a final volume of 7.5 mL and centrifuged to separate the iDNA (i.e., cell pellet) from the eDNA (i.e., supernatant). Because we did not filter the eDNA supernatant at 0.2 μm, we acknowledge that viral DNA could be found in our eDNA extracts, albeit as a minor fraction since we did not use an adapted protocol to lyse viral capsids (Danovaro and Middelboe, 2010).

Although our protocol does not specifically reduce ferric iron phases, desorption of nucleic acids from mineral surfaces is improved by the alkalinity and the phosphate content of the Na-P-buffer (Ceccherini et al., 2009). The recovery efficiency of eDNA was previously quantified by the addition of *Escherichia coli* DNA fragments of different sizes (i.e., 319, 1465, and 10 ka bp) to the Na-P-buffer and resulted in recovery rates of 77, 80, and 90%, respectively. Cumulative recovery over four washing cycles processed on three different types of sediment (i.e., Baltic Sea, Barents Sea, South Pacific Gyre) showed that 80–90% of the total volume of eDNA is efficiently extracted with three cycles, subsequent washings only produced minor quantities of eDNA (Alawi et al., 2014). We therefore performed three washing cycles with Na-P-buffer. Potential cell disruption was also tested via cell-spiking (Alawi et al., 2014).

In order to improve iDNA yield, we lysed the cell pellets prior to DNA extraction via two heating steps of 5 min at 70°C and then processed iDNA and eDNA solutions in a similar way. Each solution was mixed with three times its volume of 6 M guanidine hydrochloride and a suspension of silica particle was added to the solution to adsorb the iDNA (50 μL) and eDNA (60 μL) onto the particles. After centrifugation, supernatants were discarded and the DNA-containing silica pellets rinsed in 150 μL of equal amounts of absolute ethanol and Tris-EDTA, centrifuged twice to dry it completely. To desorb the DNA, 150 μL of Tris-EDTA buffer (1 mM) were added to the silica pellets, vortexed and centrifuged. Supernatants containing the final DNA extracts were decanted off and stored. The whole operation was repeated once, reaching final volumes of 150 and 300 μL for the iDNA and eDNA extracts, respectively (Vuillemin et al., 2016b). Final DNA concentrations were measured using a Qubit 2.0 fluorometer (Invitrogen) with 10 μL of DNA template, 1 μL of reagent and 190 μL of buffer solution, following the manufacturer’s instructions. In all cases, specificity of the eDNA and iDNA pools was checked on agarose gel and via denaturing gradient gel electrophoresis prior to sequencing (Vuillemin et al., 2016b; Supplementary Material).

### PCR Amplification for High-Throughput Sequencing

For further analysis, we selected eDNA and iDNA samples from four different sediment depths at each coring site (shallow site: 0.5, 7.5, 15, 27.5 cm depth; deep site: 1.5, 7.5, 15, 32.5 cm depth). Prior to the PCR procedure, DNA extracts were purified and eluted in a final volume of 100 μL, using a High Pure PCR Cleanup Micro Kit (Roche Applied Science). PCR was performed on both eDNA and iDNA extracts using the universal bacterial and archaeal primer pair 515F (5′-GTG CCA GCM GCC GCG GTA A-3′) and 806R (5′-GGA CTA CHV GGG TWT CTA AT-3′) with individual tags composed of 8 nucleotides at each primer 5′-end to enable multiplexing of all PCR products in a single library. PCR reactions were performed according to previously published mixtures and conditions (Lejzerowicz et al., 2013; Pawlowski et al., 2014) with eDNA and iDNA templates diluted 20 and 10 times, respectively. Negative and positive controls were added to all PCR sets using 2.5 μL of molecular grade water and 2.5 μL of *E. coli* (0.1 ng μL^-1^) as template to provide a contamination check. For each sample, 116 μL of PCR product were pooled, purified and eluted in a final volume of 25 μL. Concentrations were quantified by fluorometric method and normalized to 32 ng for each sample. Volume of pooled samples was reduced to 120 μL using a Savant SpeedVac High Capacity Concentrator (Thermo Fisher Scientific).

### Illumina Library Preparation, Sequencing, and Data Analysis

We used 60 μL of pooled PCR products (ca. 800 ng DNA) for the construction of an Illumina MiSeq library using an Illumina TruSeq DNA PCR-Free L Kit following the manufacturer’s instructions. The library was validated by qPCR using the KAPA Library Quantification Kit (Kapa Biosystems) following the manufacturer’s manual. Final concentration was quantified by fluorometric method. A MiSeq Reagent Nano kit v2, with 500 cycles with nano flow cells was used to run the library on the Illumina MiSeq Sequencing System. Two 250 cycles were used for an expected output of 500 Mb and an expected number of 7 million reads.

Quality of the raw data was checked using FastQC^2^. Demultiplexing was performed using in house scripts based on cutadapt (Martin, 2011). No errors in barcodes were allowed with Phred-Score above Q25. Read pairs were merged using PEAR (Zhang et al., 2014). Sequences were trimmed using Trimmomatic (Bolger et al., 2014). Chimeras were detected and removed using usearch61 and the ChimeraSlayer reference database (Edgar, 2010) as it is implemented in the QIIME-pipeline (Caporaso et al., 2010). OTUs were picked using the QIIME script (pick_open_reference.py), sequences were clustered and taxonomies assigned based on the SILVA database at 97% identity cut-off value (DeSantis et al., 2006). The resulting operational taxonomic unit (OTU) table was filtered by removing all OTUs with abundance below 0.1% within the sample. Sequencing data after demultiplexing were submitted to the European Nucleotide Archive^3^ under study accession number PRJEB14484.

### Statistical and Phylogenetic Analysis

For statistical analysis, duplicates were merged by the relative mean abundance of the OTUs. Non-metric dimensional scaling (NMDS) was calculated using the Past 3.10 software applying the Bray–Curtis dissimilarity index (Hammer et al., 2001). The NMDS was performed with 16 merged samples corresponding to eDNA and iDNA samples from four different sediment depths at the shallow and deep site. The NMDS was also calculated for duplicate samples (Supplementary Material).

Representative sequences were extracted for all OTUs specific to the eDNA and not found as iDNA. The SINA online v.1.2.11 aligner (Pruesse et al., 2007, 2012) was used to align our sequences. Phylogenetic analysis was performed with the ARB software package (Ludwig et al., 2004) based on the upload sequence alignments against the SILVA 16S rRNA SSU NR99 128 reference database release 07_09_2016 (Quast et al., 2013). Their closest environmental sequences and cultured species were selected as taxonomic references and used to calculate a bacterial and archaeal phylogenetic tree with nearly full-length sequences (>1400 bp) using the implemented bacterial and archaeal filter and the Maximum Likelihood algorithm RAxML (Stamatakis, 2006). Partial sequences were added to the trees using the maximum parsimony algorithm without allowing changes of tree topology. Each phylogenetic tree included representative sequences from Lake Towuti and their respective reference sequences for *Archaea* and *Bacteria*.

The GC content of each representative sequence was calculated using the MOTHUR platform (Schloss et al., 2009). To provide GC content on the class level, we normalized each value by the number of reads of the corresponding OTU and calculated weighted arithmetic means.

## Results

### DNA Concentrations, Relative Taxa Abundances, and Non-metric Dimensional Scaling

**FIGURE 2:**
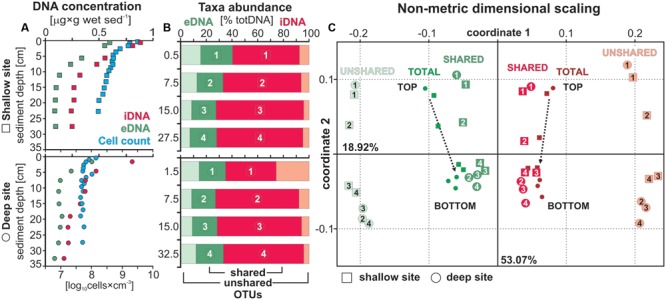
Extra- and intracellular DNA concentrations with total cell counts, taxa relative abundance and beta diversity. **(A)** DNA concentrations for the extracellular (green) and intracellular (red) fraction displayed in [μg × g wet sed^-1^] with total cell counts (blue) plotted in parallel [log_10_ cells × cm^-3^] for sediment samples at the shallow (squares) and deep (dots) site. **(B)** Relative abundances of OTUs shared (dark green, dark red) and unshared (light green, light red) by the extra- and intracellular DNA pools displayed in [% total DNA]. Each sample depth is denoted by a number and plotted as such in the following diagram. **(C)** Non-metrical dimensional scaling (NMDS) plotted for both sites emphasizing the distance between unshared eDNA and iDNA taxa. Samples related to the total eDNA and iDNA merge toward the center with sample depth as the likely result of extracellular DNA inputs due to cell lysis.

Concentrations of eDNA (**Figure 2A**) are very similar at both sites, with concentrations around 0.6 to 0.5 μg g^-1^ in the uppermost layer decreasing to ca. 0.3 μg g^-1^ at 5 cm depth and a final decline to minimum values (<0.1 μg g^-1^) at the bottom of the cores. Concentrations of iDNA parallel those of eDNA, with concentrations of 0.9 to 0.8 μg g^-1^ in the surface layer and 0.4 to 0.3 μg g^-1^ in the bottom layer for the shallow and deep site, respectively. Profiles of eDNA and iDNA concentrations display similar trends and show that iDNA is the dominant fraction (ca. 60–75%) in total DNA. The relationship between the extra- and intracellular DNA pool appears linear, whereas the relationship between total cell counts and iDNA concentrations varies between the two sites. Particularly at the shallow site, high cell densities would require additional washes of the sediments to increase cell recovery (Supplementary Material).

Alawi et al. (2014) showed that ca. 50% of the total volume of eDNA is efficiently extracted during the first washing. Total eDNA values for three different types of sediment (i.e., Baltic Sea, Barents Sea, South Pacific Gyre) scaled from ca. 780 down to 23 ng g^-1^, respectively. Values for Lake Towuti’s sediment scale from 598 to 97 and 432 to 88 ng g^-1^ at the shallow and deep site, respectively, and indicate efficient recovery after a single washing. However, due to case-by-case variations between published extraction protocols (Corinaldesi et al., 2005; Ceccherini et al., 2009; Alawi et al., 2014), eDNA concentrations have to be considered as semi-quantitative.

Overall, total cell counts (**Figure 2A**) were an order of magnitude higher at the shallow site than at the deep site. At both sites, numbers declined steeply in the upper 5 cm, followed by a more gradual decrease over the remainder of the core. Cell concentrations at the shallow site were up to 31 times higher in the 0–1 cm depth interval (i.e., mean log_10_ = 9.57 ± 0.04 and 8.52 ± 0.23) and still up to three times higher at the base of the cores (i.e., mean log_10_ = 8.21 ± 0.08 and 7.70 ± 0.02).

As regards sequencing depths, numbers of reads after chimera removal reached values between ca. 65’000 and 125’000 for the eDNA and ca. 115’000 and 158’000 for the iDNA. The corresponding numbers of OTUs were in between ca. 170 and 310, and ca. 150 and 240 for the eDNA and iDNA, respectively (Supplementary Material).

To provide comparison with an extraction approach based on tDNA (i.e., standard commercial kits), we recalculated eDNA and iDNA from their concentrations in μg g^-1^ to percentages of tDNA. We then reported the overall respective relative abundances of OTUs shared and unshared by the two pools for each respective fraction (**Figure 2B**). Relative abundances of eDNA-specific taxa (i.e., unshared OTUs) corresponded to 43 and 52% of all eDNA taxa (17–20% tDNA) decreasing to 20 and 25% (6–8% tDNA) from top to bottom of the cores at the shallow and deep site, respectively. One can thus expect to recover ca. 20% of the taxa in tDNA that either originate exogenously to the sediment or from the decay of endogenous taxa no longer detected in the sediment. These eDNA-specific taxa thus have no direct link to microbial populations presently growing in the sediment, which would otherwise be identified as taxa shared between the eDNA and iDNA pools. The relative abundance of shared eDNA taxa appears to be constant around 20%.

The NMDS calculation was based on a total of 978 OTUs corresponding to 269 OTUs shared between eDNA and iDNA, and 417 and 292 OTUs specific to the eDNA and iDNA pool, respectively. The NMDS plot (**Figure 2C**) could explain ca. 72% of the distance between samples, with coordinates 1 and 2 corresponding to 53.05 and 18.92%, respectively. Shallow and deep site samples plot very similarly. The distance plot shows that total eDNA samples are highly influenced by its OTUs that are common to the corresponding iDNA pools as their respective samples plot closer to each other with depth. On the contrary, the trend for unshared eDNA samples remain linear. For the NMDS calculated on duplicate samples, coordinates 1 and 2 explained ca. 52 and 34%, respectively (Supplementary Material).

### Relative Abundance of OTUs Shared by the Intra- and Extracellular Fraction

**FIGURE 3:**
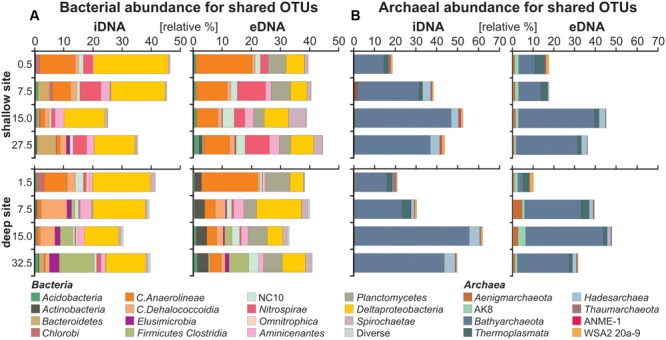
Relative abundances of bacterial and archaeal OTUs shared by both intra- and extracellular fractions. **(A)** Bacterial bar charts plotted for the iDNA and eDNA in parallel used to infer growth, decline or dormancy of certain populations (iDNA), as well as cell lysis and sequence turnover (eDNA) with depth. **(B)** Archaeal bar charts plotted for the iDNA and eDNA in parallel.

Bar charts for taxa corresponding to OTUs shared by the iDNA and eDNA (**Figure 3**) were plotted in parallel to infer, on the one hand, growth or decline of certain populations (i.e., iDNA) and, on the other hand, autochthonous inputs due to cell lysis and sequence turnover (i.e., eDNA) with depth. We acknowledge that this iDNA-based approach does not account for the degree of activity of different populations or the accumulation of intact dead cells in the sediment.

Bar charts for bacterial iDNA taxa (**Figure 3A**) show relative abundances of *Anaerolineae* and candidate NC10 decreasing with depth, whereas *Dehalococcoidia, Nitrospirae*, and *Aminicenantes* first increase and then decrease with depth. These populations were considered in decline with depth. *Deltaproteobacteria* have a rather constant relative abundance with depth, while *Clostridia* and *Elusimicrobia* increase gradually with sediment depth at the deep site, indicating that these populations likely grow with depth. In comparison, bar charts plotted for bacterial eDNA taxa shared with the iDNA show that abundances of the same taxa tend to covary between iDNA and eDNA pools. Abundances of *Planctomycetes* and *Actinobacteria* are substantial in the eDNA and very minor in the iDNA, in which they can account for the presence of persistent cells.

Bar charts for archaeal iDNA taxa (**Figure 3B**) reveal a dominance of *Bathyarchaeota* with an increase from ca. 15 to 50% of all taxa recovered in the iDNA pool with depth. To a much lesser extent *Hadesarchaea* also increase with depth. *Thermoplasmata* and *Aenigmarcheaota* decrease with depth, while candidates ANME-1 and WSA2 are minor. Bar charts plotted for archaeal eDNA taxa display similar trends to the iDNA though minor taxa tend to diverge between the two pools. Abundances of *Bathyarchaeota* and *Hadesarchaea* increase proportionally to those of the iDNA. *Thermoplasmata* decrease with depth, *Aenigmarcheaota* and candidate AK8 are constant at the shallow site, but fluctuate with depth at the deep site. These latter taxa are mainly found in the eDNA pool.

### Relative Abundances of Unshared eDNA Taxa and Their Phylogenetic Affiliations

**FIGURE 4:**
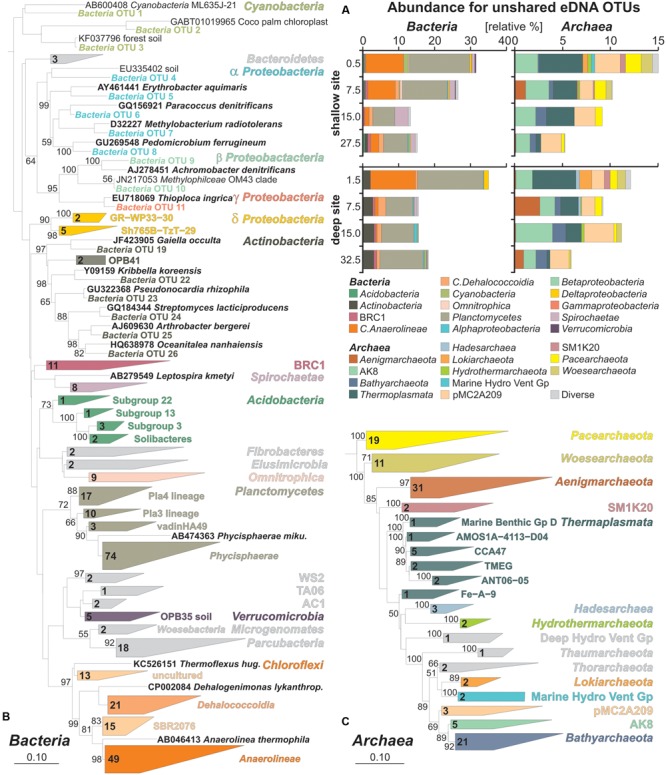
Relative abundances of bacterial and archaeal OTUs specific to extracellular DNA and phylogenetic trees based on their partial 16S rRNA gene sequences. **(A)** Bacterial and archaeal bar charts plotted for taxa based on OTUs specific to the unshared eDNA. **(B)** Maximum likelihood tree with bootstrap values established for representative bacterial OTU sequences exclusive to the eDNA with database references and accession numbers in boldface types. **(C)** Maximum likelihood tree established for representative archaeal OTU sequences exclusive to the eDNA with database references and accession numbers in boldface types.

Bar charts of bacterial OTUs exclusive to the eDNA pool (**Figure 4A**) show a majority of *Anaerolineae*, whose abundance decreases with depth, and *Planctomycetes* which remain nearly constant. *Actinobacteria* are mainly present at the deep site. At the shallow site, the abundance of *Spirochaetae* increases with depth. Minor taxa include *Cyanobacteria, Alpha-, Beta-, Delta-*, and *Gammaproteobacteria* among others. Bar charts for unshared eDNA archaea reveal a decrease of *Thermoplasmata, Pacearchaeota, Woesearchaeota* and candidate SM1K20 with depth. Candidate pMC2A209 and AK8 remain rather constant with depth. *Bathyarchaeota* are also present in the unshared eDNA and slightly increase with depth at both sites, whereas the presence of *Hadesarchaea* is minor and restricted to surface sediments of the deep site. Similarly, *Lokiarchaeota* are exclusively found in uppermost sediments (**Figure 4A**).

Representative sequences were extracted for all 417 OTUs of the unshared eDNA pool and plotted into two separate phylogenetic trees for *Bacteria* and *Archaea*. Results of bacterial phylogeny consist of 300 OTUs from Lake Towuti and their closest match sequences in the SILVA database (**Figure 4B**). *Cyanobacteria* include sequences of planktonic species as well as chloroplasts. Closest matches among *Proteobacteria* are related to organisms with known or implied capacity to grow through iron and sulfur oxidation, nitrate and sulfate reduction, as well as some through methylotrophy. *Actinobacteria* and *Verrucomicrobia* mainly correspond to taxa-related organisms commonly found in soils. *Planctomycetes* are mostly affiliated with the *Phycisphaerae*, although three other lineages were resolved. Among the *Chloroflexi, Anaerolineae* are the most numerous followed by *Dehalococcoidia*, candidates SBR2076 and some other currently uncultured taxa. *Acidobacteria* were resolved as four subgroups, including *Solibacteres* that are typically found in soils, but altogether are a minor component of the eDNA taxa. Bacterial sequences with unassigned taxonomies were resolved in the ARB database as candidates WS2, TA06, AC1, *Microgenomates* and *Parcubacteria.* Additional minor groups include candidate BRC1*, Omnitrophica, Bacteroidetes, Fibrobacteres*, and *Elusimicrobia*.

The archaeal phylogenetic tree includes 117 OTUs from Lake Towuti and their closest matched sequences from the SILVA database (**Figure 4C**). All archaeal sequences lacked closely related cultivated representatives and phylogenetic affiliations mainly correspond to candidate divisions of the TACK (i.e., *Thaumarchaeota, Aigarchaeota, Crenarchaeota*, and *Korarchaeota*) and DPANN (i.e., *Diapherotrites, Parvarchaeota, Aenigmarchaeota, Nanoarchaeota*, and *Nanohaloarchaeota*) supergroups (Castelle et al., 2015; Solden et al., 2016) with the exception of *Thermoplasmata*. In terms of number of representative OTUs, *Aenigmarchaeota, Bathyarchaeota, Pacearchaeota*, and *Woesearchaeota* are the most numerous. *Thermoplasmata* were resolved in six different clusters, CCA47 being the most represented. Members of the candidate phyla AK8 and pMC2A209, although relatively abundant, comprise a limited number of OTUs. Diverse but minor groups include *Hadesarchaea*, candidate SM1K20, *Thorarchaeota, Lokiarchaeota, Hydrothermarchaeota, Thaumarchaeota*, and the Marine Hydrothermal Vent and Deep Hydrothermal Vent Groups.

### GC Content of Unshared eDNA Sequences

**FIGURE 5:**
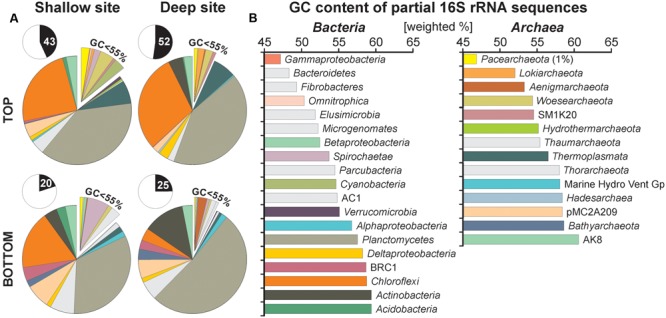
Pie charts for taxa specific to the unshared extracellular DNA and GC contents of their partial 16S rRNA gene sequences. **(A)** Pie charts plotted for unshared eDNA taxa from core top and bottom samples of the shallow and deep site. Inserts indicate abundances of unshared eDNA relative to total eDNA. **(B)** GC contents of partial 16S rRNA sequences calculated on the class level based on 417 representative sequences and displayed in [weighted %]. Although initial sizes of each population are determinant at the time of burial, most sequences composing the unshared eDNA have GC contents above 55%, arguing for preferential preservation.

To trace preferential preservation of sequences that can potentially occur with depth, we extracted GC contents of partial 16S rRNA sequences for all 417 unshared eDNA representatives. Relative abundances for unshared eDNA taxa are displayed on the pie charts according to the GC content of their partial 16 rRNA gene sequences, which increase in the clockwise direction (**Figure 5A**). We averaged values by the relative abundance of each OTU sequences in order to list GC contents on the class level (**Figure 5B**). Bacterial classes displaying high GC contents (>55%) (Galtier et al., 1999) are *Verrucomicrobia* (55.1%), *Alphaproteobacteria* (56.7%), *Planctomycetes* (57.5%), *Deltaproteobacteria* (58.2%), candidate BRC1 (58.7%), *Chloroflexi* (58.7%), *Actinobacteria* (59.3%), and *Acidobacteria* (59.3%). Similarly, archaeal classes with high GC contents are *Hydrothermarchaeota* (55.1%), *Thaumarchaeota* (55.4%), *Thermoplasmata* (56.5%), *Thorarchaeota* (58.0%), Marine Hydrothermal Vent Group (58.1%), *Hadesarchaea* (58.4%), candidate pMC2A208 (58.4%), *Bathyarchaeota* (58.6%), and candidate AK8 (60.6%).

Taxa found to be abundant in the unshared eDNA fraction have generally GC contents above 55% with relative abundances kept constant with depth. Sequences of *Planctomycetes, Actinobacteria*, and *Bathyarchaeota* appear to be best preserved with depth, whereas those of *Thermoplasmata* and *Chloroflexi* decrease gradually (**Figure 5A**). In comparison, relative abundances of classes with lower GC contents, such as *Cyanobacteria, Pacearchaeota*, and *Woesearchaeota* tend to decline with depth.

## Discussion

### Tracing Allochthonous DNA Sources

Environmental DNA preserved in lacustrine sediments contains information of past environmental conditions (Coolen et al., 2008; Boere et al., 2009, 2011a; Anderson-Carpenter et al., 2011; Vuillemin et al., 2014a; Ariztegui et al., 2015). However, the composition of buried eDNA results from a combination of sediment provenance, lacustrine conditions at the time of deposition and microbial growth and activity within sediments (Torti et al., 2015; Vuillemin et al., 2016a). This confounds the direct inference of paleoconditions from eDNA. Nevertheless, environmental dynamics like changing river discharge or water column structure have a direct influence on the making of sedimentary eDNA and, in Lake Towuti, may result in the divergence of eDNA compositions between the shallow and deep site.

For example, terrestrial sediment from the catchment is transported to Lake Towuti by the Mahalona River through the distal part of the Tominanga Delta and from there into the deep basin (**Figure 1B**). This mainly affects sedimentation at the deep site (Vogel et al., 2015). In contrast, shorter settling times at the shallow site (i.e., 60 m vs. 200 m depth at the deep site) likely increase the chances of preserving eDNA from primary producers in the shallow site sediments (i.e., 60 m depth). Furthermore, the water column is presently stratified with oxygen depletion below ca. 130 m water depth (Vuillemin et al., 2016b). Such chemical stratification drives a succession of microbial functional guilds with water depth in accordance with the availability of respiratory electron acceptors and donors. The sedimentation flux of pelagic cellular material to the shallow and deep site is thus likely taxonomically distinct. For these reasons the shallow and deep coring sites should contain different eDNA assemblages reflecting their respective depositional features.

Environmental sequences belonging to taxa exclusively found in the eDNA pool were considered exogenous to the sediment and represented more than 40–50% of total eDNA taxa in surface sediments at both sites (**Figures 4A, 5A**). In terms of sediment provenance, terrestrial material transported by the Mahalona River resulted in several sequences related to soil environments at the deep site, which included a majority of *Actinobacteria* and some *Verrucomicrobia, Solibacteres* and *Alphaproteobacteria*, e.g., *Pseudonocardia, Pedomicrobium* (Janssen et al., 2002). At the shallow site, sequences derived from soils were also identified, mainly as Malaysian soil-related *Spirochaetae* (Slack et al., 2009), plus few *Verrucomicrobia* and *Cyanobacteria* from forest soil and palm tree chloroplast, which indicates an increasing anthropogenic influence due to land use in this area (Parenti and Soeroto, 2004). Because of phosphorus limitation in the water column, sequences of prokaryotic primary producers were rare among the unshared eDNA. The presence of *Cyanobacteria* was minor at the shallow site, while sediments of the deep site contained some sequences of *Chlorobi* candidates, mainly preserved in the iDNA (**Figure 3A**). *Chlorobi*, also known as green sulfur bacteria, are mostly obligate anaerobic phototrophs (Manske et al., 2005) which could be indicative of past stratification and photic zone euxinia (Coolen and Overmann, 2007; Crowe et al., 2008a). However, their presence in the iDNA and phylogenetic relationship to *Ignavibacteria* presently link them to anaerobic chemoheterotrophs (Iino et al., 2010). Due to non-sulfidic conditions, purple sulfur bacteria were likewise absent (Coolen and Overmann, 1998; Bosshard et al., 2000). Concerning an eventual anoxic photic zone in the water column, *Anaerolineae*-related *Chloroflexi*, whose abundances are substantial in surface sediments at both sites, were thought to perform photoheterotrophy (Liu et al., 2011). However, it appears that their biosynthesis of bacteriochlorophyll is incomplete but still represents a potential for mixotrophic growth in the near-infrared (Klatt et al., 2013). These *Anaerolineae*-related sequences are indicative of heterotrophy by facultative anaerobes (Sekiguchi et al., 2003) and, theoretically, limited light penetration in the anoxic hypolimnion. Major differences between the two studied sites are *Actinobacteria* and *Chloroflexi* abundances, which were respectively higher and rapidly decreasing with depth at the deep site. Thus, in contrast with Lake Matano (Crowe et al., 2008a), modern Lake Towuti does not display clear features of an anoxic photic zone.

In addition, various eDNA sequences related to both aerobic and anaerobic heterotrophs point toward bacterial secondary production in the stratified water column. Among sequences of *Proteobacteria*, we detected potential types of respiration involving aerobic processes of manganese (α) and sulfur (γ) oxidation and anaerobic processes of nitrate (α, β) and sulfate (δ) reduction (**Figure 4B**). Reference sequences for *Alpha-* and *Betaproteobacteria* matched aerobic and anaerobic methylotrophs and further included candidates among *Thermoplasmata* potentially remineralizing organic matter to methane (Poulsen et al., 2013). Other likely anaerobic fermenters included candidates BRC1, WS2, TAO6, and AC1 (Chouari et al., 2005). However, most candidate taxa were difficult to associate with any specific environmental feature (Solden et al., 2016). *Pacearchaeota* and *Woesearchaeota* were found to thrive in surface water of oligotrophic lakes (Ortiz-Alvarez and Casamayor, 2016), which is consistent with present-day lake conditions, whereas the lifestyle of *Parcubacteria* involves ectosymbiosis or parasitism of other organisms (Nelson and Stegen, 2015). Nevertheless, most heterotrophic sequences in the unshared eDNA were affiliated with facultative anaerobes such as *Phycisphaerae* (Fukunaga et al., 2009) and *Anaerolineae* (Sekiguchi et al., 2003). Such microorganisms are characteristic of anoxic aquatic and sediment habitats and common in microbial mats often persisting long after the mat degradation (Lage and Bondoso, 2014). They appear to be responsible for quick degradation of sinking particulate organic matter and planktonic sequences at the water-sediment interface, resulting in their preferential preservation during early phase of burial (Cole et al., 2014).

Present observations demonstrate that eDNA sequences record specific lacustrine features such as river inflow, trophic state and stratification of the water column. However, such recording requires preservation of initial sources which appears to be a function of sedimentation type and secondary production rate.

### Former and Current Microbial Sequences Endogenous to the Sediment

Below the water-sediment interface, the concentration and composition of eDNA can rapidly be modified by growth and decline of microbial populations in the uppermost sediment layers (**Figure 2A**). The release of eDNA upon cell lysis gives rise to an accumulation of short nucleic fragments with sediment depth that can rapidly overlap with and cover the previous eDNA composition during early diagenesis (Vuillemin et al., 2016a). Subsequent to burial, persisting activity of certain microbial populations determines the recording of sedimentary eDNA as a function of sequence degradation and turnover (Boere et al., 2011a; Corinaldesi et al., 2011). In order to constrain these factors in our interpretation of eDNA sequences, we used relative taxa abundances in the shared iDNA pool to deduce which populations tend to grow or decline with depth. We acknowledge that inactive intact cells can nevertheless accumulate within sediments.

On the basis of shared iDNA abundances (**Figure 3**), microbial populations seemingly growing with sediment depth consisted of *Deltaproteobacteria, Clostridia, Elusimicrobia, Bathyarchaeota*, and *Hadesarchaea*. Growing and subsequently declining populations comprised *Dehalococcoidia, Nitrospirae, Aminicenantes, Aenigmarchaeota*, and *Thermoplasmata*. Populations in decline from the water-sediment interface included *Chlorobi, Anaerolineae, Omnitrophica*, and candidate NC10. *Planctomycetes, Actinobacteria*, and *Acidobacteria* appeared to be dormant and were mainly present as eDNA. We considered that this succession of microbial populations reflected the geochemical and organic evolution of the sediment during early diagenesis (Vuillemin et al., 2016b). Cell lysis as an endogenous source of eDNA was clearly linked to populations whose relative abundances increased and resulted in an accumulation of the corresponding sequences with depth (**Figure 3**). Populations displaying abundances in decline tended to be gradually erased from the eDNA record, thus pointing at a proportional link in abundances of taxa found as iDNA and eDNA. Stable and scarce populations could represent the ability to enter into a dormant (non-dividing) state and the presence of persistent cells in the sediment (Lewis, 2007; Starnawski et al., 2017). Their respective abundance in the eDNA appeared to be kept constant or to even increase with depth, which could indicate enhanced preservation in biofilms at the time of burial (Lage and Bondoso, 2014). This shows that sedimentary eDNA is constituted by microbial elements preserved differentially during early diagenesis (Boere et al., 2011a; Corinaldesi et al., 2011; Vuillemin et al., 2016a). For instance, sequences presently related to known spore- and cyst-formers, including *Actinobacteria, Clostridia*, and *Planctomycetes*, were better preserved with depth (**Figure 5A**). This also makes it clear that the eDNA fraction, whether it be exogenous or endogenous to the sediment, can bias microbial community studies based on tDNA toward higher diversity (**Figure 2B**) (Luna et al., 2006; Alawi et al., 2014; Carini et al., 2016).

Persistence of dormant populations has further implications in terms of activity and diversity of environmental taxa in long sedimentary records (Johnson et al., 2007; Lennon and Jones, 2011) since their dispersal and viability could complicate source assignment (Lozupone and Knight, 2007). For instance, DNA inside resting stages of certain soil-related or planktonic species (e.g., cysts, endospores, and akinetes) can be better preserved than DNA from microbial taxa that do not produce such resistant life stages (Willerslev et al., 2004; Coolen and Overmann, 2007; Anderson-Carpenter et al., 2011). In surface sediment, a small fraction of these communities can integrate the subsurface assembly and even persist at reduced growth rates (Starnawski et al., 2017). The successive activities of subsurface populations thus undermine the temporal relationship between stratigraphic deposition of the sediment and its iDNA composition. Comparison with the eDNA then allows the discrimination of ancient and persistent taxa derived from erosion, in-lake and post-depositional processes. Preserved fossil genes are used to trace successions of primary assemblages in stratigraphic intervals when standard paleoecological indicators are degraded, dissolved or not specific enough (Boere et al., 2009; Coolen et al., 2009; Lejzerowicz et al., 2013). In the case of thorough environmental studies, it requires stringent extraction protocol lysing all cells in order to recover the entire pool of ancient DNA and specific primer pairs to improve detection limits (Coolen, 2011; Coolen et al., 2013). The same applies to ancient 18S rRNA sequences (e.g., seeds, pollens, dinocysts, and frustules) (Parducci et al., 2005, 2017; Boere et al., 2011b).

### Preferential Turnover of Extracellular DNA and its Implications

Prior to sediment diagenesis, the ability to adapt to changing environmental conditions controls initial growth and sizes of microbial populations. Subsequent factors known to influence the fate of eDNA in the sedimentary record include processes like microbial activity, adsorption to mineral and organic matrices, as well as various physical conditions such as temperature and salinity (Levy-Booth et al., 2007). With regards to physical factors, temperature in the water column and sediment of Lake Towuti is 31–28°C throughout the year with freshwater and phosphorus-limited conditions, which do not enhance eDNA preservation (Lindahl, 1993). In addition, the water column and sediment are slightly alkaline (pH = 8.4 to 7.2), whereas DNA adsorption is promoted at lower pH (Levy-Booth et al., 2007).

Exogenous sources of eDNA to the sediment were successively related to soils reworked from the ferruginous catchment (i.e., *Actinobacteria, Verrucomicrobia, Acidobacteria*) and only a very limited number of sequences arising from primary producers (i.e., *Cyanobacteria*). A first selective degradation by heterotrophs occurred in the water column (i.e., *Proteobacteria, Chloroflexi, Thermoplasmata*) and at the water-sediment interface (i.e., *Planctomycetes, Chloroflexi*). Endogenous sources of eDNA then comprised a succession of microbial populations related to geochemical evolution of the sediment during early diagenesis (i.e., *Dehalococcoidia, Deltaproteobacteria, Bathyarchaeota, Hadesarchaea*). The rapid drop in eDNA concentrations with sediment depth (**Figure 2A**) could thus result from the immediate degradation of the free eDNA along with an overall decrease in metabolic activity and cell lysis rate (Vuillemin et al., 2016b). Ferric mineral phases, which are known to persist down to 15 m sediment depth (Simister et al., 2016), were considered unlikely to hold a substantial adsorbed fraction (Ceccherini et al., 2009). Incubations of ferruginous sediments from Lake Matano showed that hydrous ferric oxides (i.e., ferrihydrite) are rapidly reduced by dissimilatory metal-reducing bacteria, resulting in the dissolution of Fe–Mn oxyhydroxides with persistence of mainly goethite (Crowe et al., 2007a,b). The affinity of DNA for goethite is substantially lower than that of common phyllosilicates (Cai et al., 2006), and because goethite traps organic molecules indifferently, its sorption capacity also decreases with increasing organic matter content and degradation (Crecchio et al., 2005). Complete reduction of ferrihydrite occurs within the first cm of sediment at the shallow site, whereas it is even absent at the deep site due to its prior reduction in the water column (data not shown). The increase of Fe^2+^ and NH_4_^+^ concentrations in pore water with sediment depth (Supplementary Material) implies the decrease of sorption capacities due to microbial activity. Finally, the lower quality of the eDNA at the shallow site (Supplementary Material) reflects the preferential adsorption of short single-stranded DNA onto ferric iron phases (Nielsen et al., 2007; Cleaves et al., 2011).

Beyond these considerations, the preservation of fossil DNA is known to differ between species and remains poorly understood (Boere et al., 2011a). Since bacterial degradation of adenosine monophosphate is faster than cytidine monophosphate (Dell’Anno et al., 2002), we expected an increase of GC content in the unshared eDNA sequences if eDNA is used and selectively degraded as a nutrient source after deposition (Dell’Anno and Danovaro, 2005). In terms of recording past environmental conditions, exogenous sources of eDNA were mainly represented by sequences of *Chloroflexi, Planctomycetes* and *Actinobacteria* among bacteria and *Thermoplasmata*, candidates pMC2A209 and AK8 among archaea (**Figure 5A**). These taxa display GC contents ranging from 57 to 60%, respectively (**Figure 5B**). Considering *Actinobacteria* and *Planctomycetes*, the total GC content of their genomes (64 and 73%) and types of cell membrane constitute additional factors explaining their preservation (Fukunaga et al., 2009; Ganzert et al., 2011). Otherwise, *Pacearchaeota* (GC: 46.8%) and *Woesearchaeota* (GC: 54.4%), which originated from the water column, were present in the uppermost sediment but rapidly decreased with sediment depth, whereas *Thermoplasmata* (GC: 56.6%) and *Chloroflexi* (GC: 58.7%) were gradually and partially lost only after the decline of their populations in the sediment. Candidate pMC2A209 (58.4%) and AK8 (GC: 60.7%) remained constant throughout the cores. Active populations, such as the *Deltaproteobacteria* (GC: 58.2%), *Hadesarchaea* (GC: 58.4%) and *Bathyarchaeota* (GC: 58.6%) which actively grow at the expenses of former and declining population sequences with lower GC contents, quickly dominate the total eDNA pool over time.

In addition, preservation of cells embedded in biofilms or entering into a persistent non-dividing state occurs during burial, thereby shaping the composition of fossil iDNA with sediment depth. Sustained activity of the subsurface biosphere in parallel establishes the final eDNA composition, whose recording gradually shifts temporally from the stratigraphic context. The extent of post-depositional degradation also limits standard PCR assays due to fragment shortening over time (Boere et al., 2011a,b). Thus, the search for fossil 16S rRNA gene sequences requires high initial amount of sediment (Coolen and Overmann, 2007) and controlled sampling to prevent cells exiting dormant state (Jones and Lennon, 2010; Starnawski et al., 2017). Primers targeting representative microorganisms can be used to decrease detection limits and collect specific information on past climatic conditions (Coolen and Overmann, 1998, 2007; Coolen et al., 2008) regardless of diagenetic modifications.

## Conclusion

In surface sediment of ferruginous Lake Towuti, the extracellular fraction accounted for ca. 40% of total extracted DNA. Environmental sequences exclusively found as eDNA were considered exogenous to the sediment and displayed an overall composition kept constant with depth. Paleoenvironmental information interpreted from these 16S rRNA sequences pointed at multiple sources foreign to the sediment, such as soil inputs reworked from the catchment, limited primary production and pronounced secondary production in the water column. The related heterotrophs reflected aerobic and anaerobic processes involving potential for methylotrophy, manganese and sulfur oxidation as well as nitrate and sulfate reduction in accordance with chemical stratification of the water body. High abundances of *Chloroflexi* and *Planctomycetes* in surface sediments argued for substantial degradation of sinking organic matter and planktonic sequences in the bottom water and at the water-sediment interface, resulting in their preferential preservation during early phase of burial. After burial, the use of eDNA as a nutrient source by active resident microbial populations led to substantial and selective degradation of sequences of lower GC contents. Cell lysis in the sediment constituted an important endogenous source of eDNA, which tended to overlap and cover prior genetic assemblages. Thus, extant populations growing with depth quickly came to dominate in the total eDNA pool at the expenses of former and declining populations. The presence of persistent cells could nevertheless be detected.

We conclude that eDNA preserved in shallow lacustrine sediments initially reflects limnological features. However, the increasing influence of a subsurface biosphere brings on modifications in its composition that shift temporally the final recording from the stratigraphic context. Separate eDNA and iDNA extractions make it possible to discriminate between sources exogenous and endogenous to the sediment and, thereby, to address in-lake and post-depositional processes in parallel. In deeper sediments, the expected accumulation of resting stages and sequences from cell lysis requires a stringent extraction and specific primer pairs if the target of research lies in the ancient DNA.

## Author Contributions

AV performed DNA extractions, Illumina sequencing procedure, genetic and statistical analyses, designed the figures and led the writing of the present manuscript. FH treated the raw sequencing data, designed and ran scripts of the pipeline and supervised genetic and statistical analyses. MA designed and supervised DNA extractions, genetic and statistical analyses. CH fulfilled the research permit procedure. DW provided important financial and technical support and supervised genetic analyses. SC sampled during field campaign and designed the study. JK designed the study, sampled during field campaign, and supervised the writing of the present manuscript. All authors have taken part in the manuscript revisions and agreed with its scientific content.

## Conflict of Interest Statement

The authors declare that the research was conducted in the absence of any commercial or financial relationships that could be construed as a potential conflict of interest.

## Footnotes

1 http://www.pangaea.de/

2 http://www.bioinformatics.babraham.ac.uk

3 http://www.ebi.ac.uk/ena
