# Supplementary material for: Preservation and Significance of Extracellular DNA in Ferruginous Sediments from Lake Towuti, Indonesia
Source: Front Microbiol. 2017 Jul 27;8:1440. doi: 10.3389/fmicb.2017.01440 (PMC5529349; doi:10.3389/fmicb.2017.01440)
Supplement: Supplementary file 1 [file Image_1.PDF]

Preservation and significance of extracellular DNA in ferruginous sediments of Lake Towuti, Indonesia

Aurèle Vuillemin, Fabian Horn, Mashal Alawi, Cynthia Henny, Dirk Wagner, Sean A. Crowe, & Jens Kallmeyer*

*** Correspondence:** kallm@gfz-potsdam.de

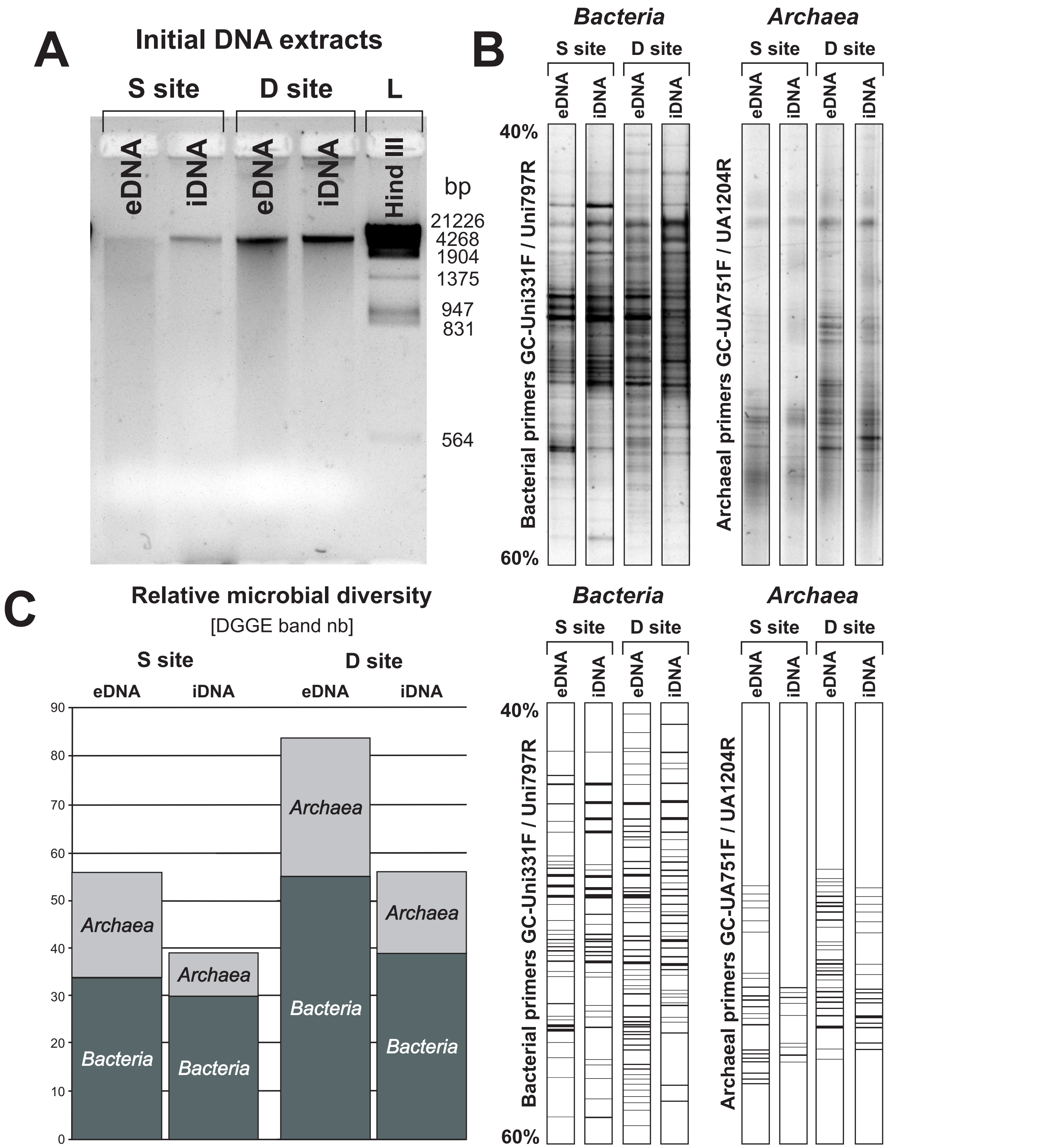

Figure S1. Agarose gel of initial DNA extracts (A), DGGE gel images (B) and comparison of their band patterns (C). The extraction protocol was prealably tested on grab sample sediments retrieved at the shallow (60 m depth) and deep site (200 m depth).DNA extracts run on agarose gel show different quality of DNA with the extracellular and intracellular fractions displaying smears and clear bands, respectively. Band patterns of DGGE gels emphasize the higher number of bands but lesser intensities of those related to extracellular DNA. Additional DGGE gel comparisons are available in Vuillemin et al. 2016 (*Front. Microbiol.* 7, e1007).

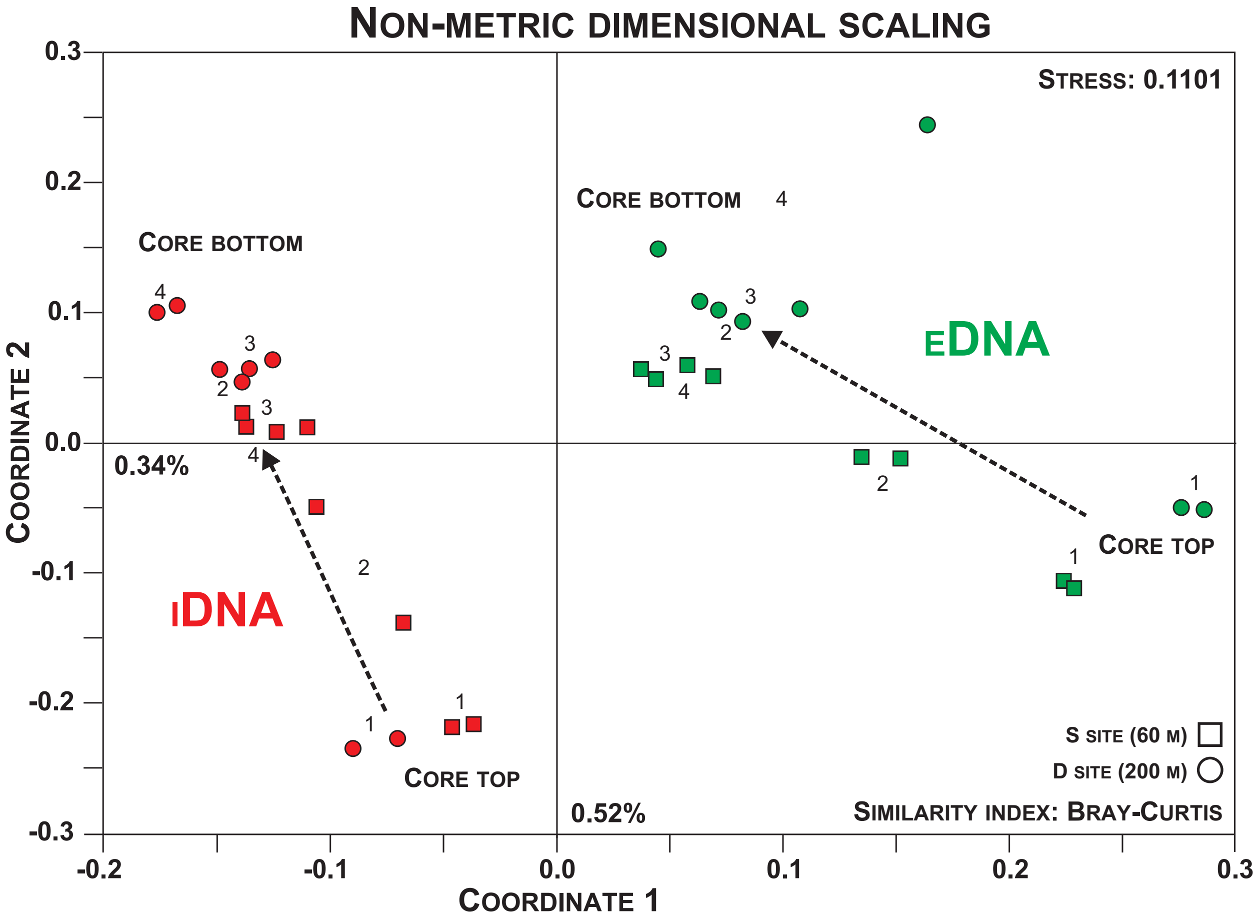

Figure S2. Plot of non-metric dimensional scaling analysis performed on duplicate samples of extra- and intracellular DNA. Results of β diversity analysis show good reproducibility of the duplicates. Distances on the plot emphasize the specificity of the intracellular (red) and extracellular (green) DNA pools, as well as the general trend with sediment depth.

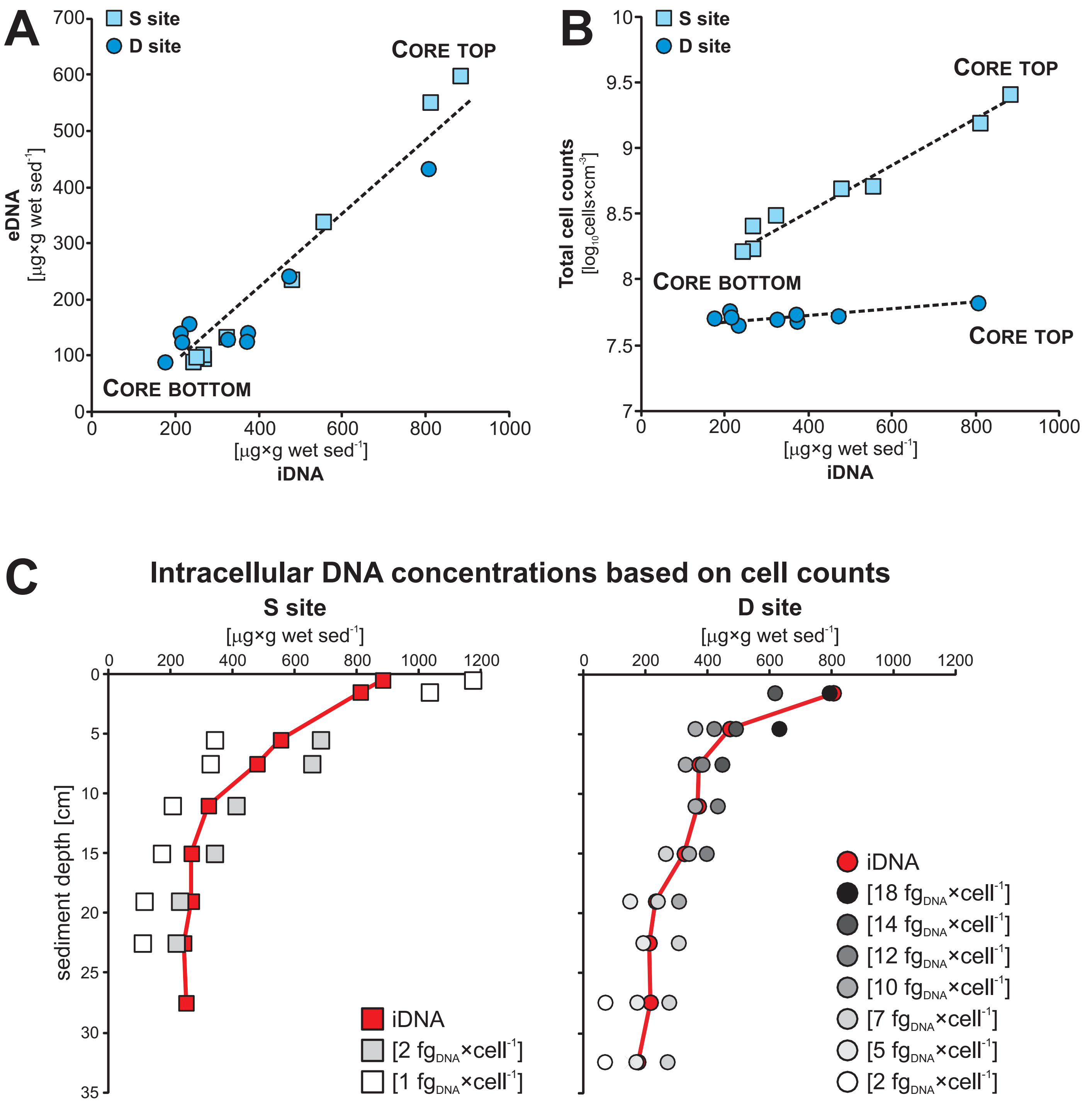

Figure S3. Scatter plots for intracellular and extracellular DNA concentrations (A), intracellular DNA concentrations and total cell counts (B), and theoretical DNA concentrations based on total cell counts (C). The scatter plot of intra- and extracellular DNA concentrations (**A**) shows that these two pools are linearly correlated.The relationship between intracellular DNA and total cell counts (**B**) appears to be exponential at the shallow site and linear at the deep site. DNA concentrations per cell necessary to equal measured ones (**C**) vary between sites and change with sediment depth. At the shallow site, they correspond to ca. 1 to 2 femtograms of DNA per cell, whereas at the deep site they decrease gradually with depth from 18 to 5 femtograms of DNA per cell. Although these concentrations are realistic, we consider the yield of intracellular DNA to be semi-quantitative and to require additional washes of the sediments to achieve quantitative cell recovery.

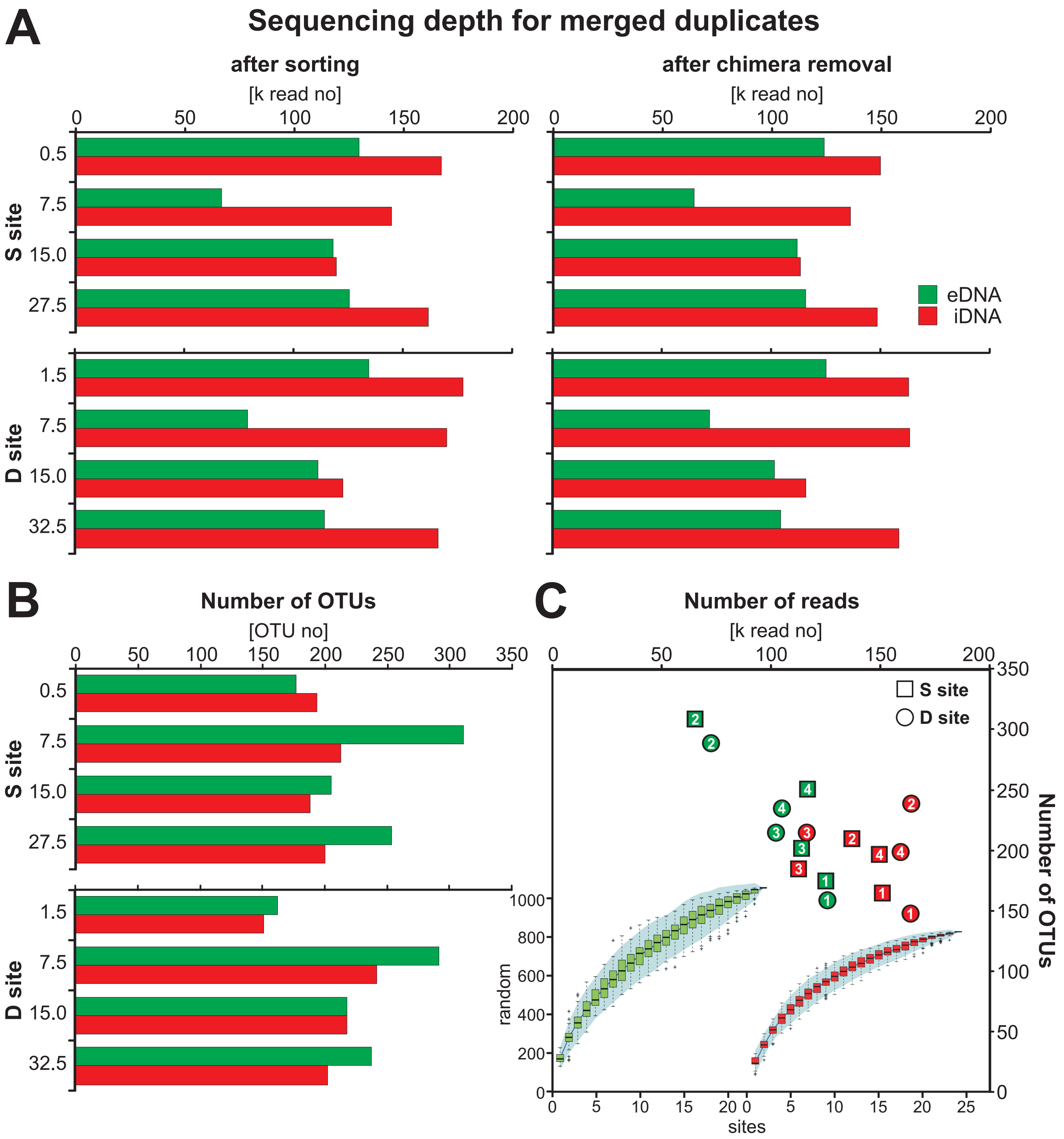

Figure S4. Bar charts displaying the number of reads for extra- and intracellular DNA (A), number of OTUs (B) and cross plot displaying the number of reads and OTUs (C). Read numbers are systematically higher for the iDNA (**A**), whereas the number of OTUs tends to be higher for the eDNA (**B**). The cross plot (**C**) shows that the second eDNA samples of both sites have the highest OTU numbers and lowest read numbers. The insert shows that species accumulation is higher for the eDNA.

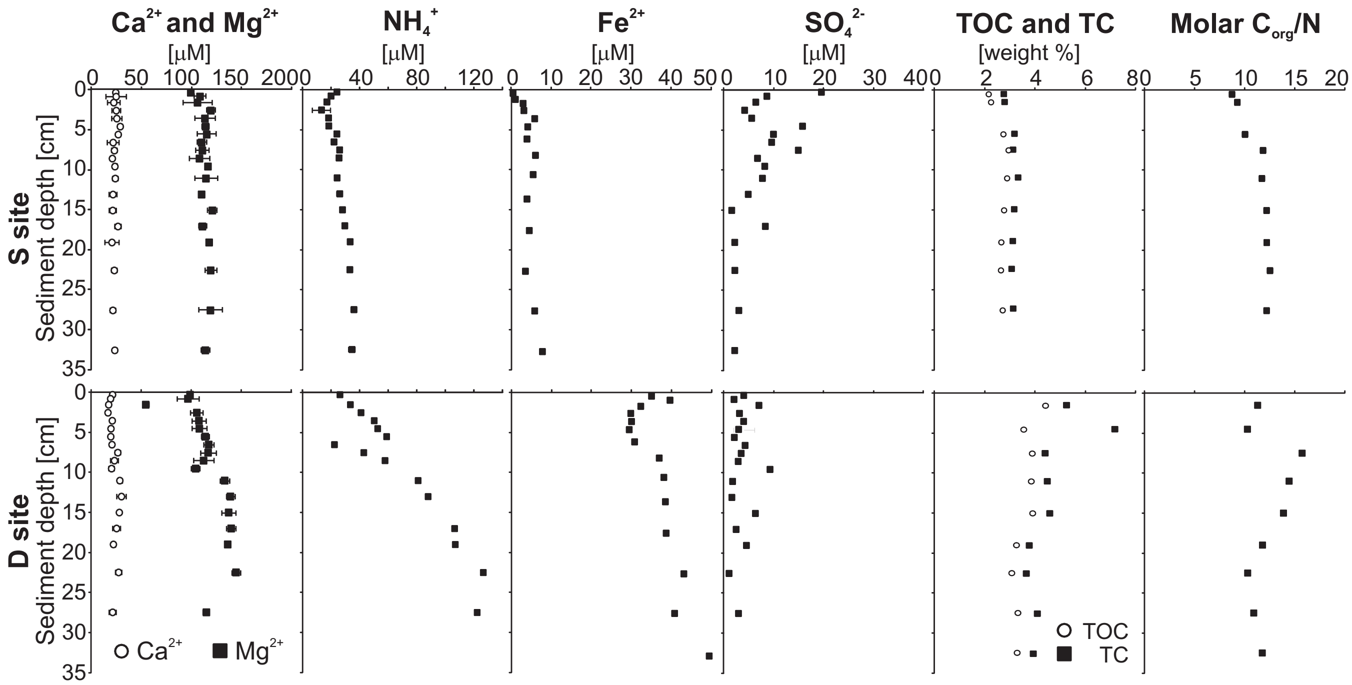

Supplementary Figure S5. Multiple sediment downcores obtained for the shallow and deep site. From left to right: Calcium, magnesium, ammonium, dissolved ferrous iron, and sulfate concentrations [µM] measured in the pore water; total organic carbon, total carbon [weight %] and molar C_{org}/N ratio measured in bulk sediment Data were previously published in Vuillemin et al. 2016 (*Front. Microbiol.* 7, e1007).
